# Screening for Mutations in Hereditary Cancer Susceptibility Genes in a Region with High Endogamy in Brazil

**DOI:** 10.1055/s-0043-1777449

**Published:** 2023-12-08

**Authors:** Polyanna Oliveira, Paula Correa, Angelina Acosta, Juliana Freitas, Taísa Machado-Lopes, Thais Bomfim-Palma, Ândrea Ribeiro-dos-Santos, Sidney Santos, Roberto Nascimento, Ivana Nascimento, Kiyoko Abe-Sandes

**Affiliations:** 1 Department of Biology, State University of Feira de Santana, Bahia, Brazil; 2 Medical Genetics Service, University Hospital Prof. Edgard Santos, Salvador, Bahia, Brazil; 3 Department of Life Sciences, State University of Bahia, Bahia, Brazil; 4 Laboratory of Immunology and Molecular Biology, Institute of Health Sciences, Federal University of Bahia, Salvador, Brazil; 5 Laboratory of Human and Medical Genetics, Institute of Biological Sciences, Federal University of Pará, Pará, Brazil

**Keywords:** hereditary cancer, pathogenic variant, multigene panel, endogamy

## Abstract

**Introduction** Cancer is a multifactorial disease dependent on the influence of genetic and environmental factors. About 10% of cancers are associated with germline mutations, which predispose to a higher risk of developing cancer. Currently, the use of panels that identify susceptibility and/or association genes cancer has been increasingly used, both in clinical practice and in scientific research.

**Objective** To investigate genetic mutations in patients with a profile for hereditary cancer in individuals from a region of northeast Brazil, where there is a high frequency of endogenous and consanguineous marriages.

**Methods** A set of 17 genes ( *BRCA1* , *BRCA2* , *APC* , *TP53* , *PTEN* , *RET* , *VHL* , *RB1* , *CDKN2* , *CDH1* , *CHEK2* , *MLH1* , *MSH2* , *MSH6* , *MUTYH* , *XPA* , and *XPC* ) associated with cancer and hereditary syndromes were analyzed. Fifteen patients with a hereditary cancer profile were evaluated.

**Results** The pathogenic variant found was c.1187G > A (p.Gly396Asp), rs36053993 in the *MUTYH* gene in a male patient diagnosed with melanoma at the age of 43 years and a family history for this tumor. This gene encodes an important enzyme related to DNA repair and has been associated with other types of cancer, this is the first report of an association with melanoma, the biological plausibility of this association is given once the MUTYH protein is expressed in the skin tissue and is responsible for repairing damage caused, for example, by sun exposure.

**Conclusion** The results of this study suggest that this mutation may be important for the hereditary predisposition to melanoma, but a broader investigation of this mutation is needed.

## Introduction

All cancers are caused by genetic material changes, but only a small proportion (5–10%) is due to inherited mutations. 1 Therefore, the identification of individuals carrying pathogenic variants in hereditary cancer susceptibility genes allows: early screening and risk reduction protocol implementation. 2

Molecular diagnostic tests for hereditary cancer have traditionally been restricted to the analysis of one or a few genes, selected from the syndrome identified in the family. 3 However, cancer susceptibility may occur due to one or more mutations in one of several different genes related to tumor development, not necessarily related to clinical suspect. 4 Due to genetic heterogeneity, the mutation responsible for the disease is not always in a gene classically related to that syndrome, as in the case of the *BRCA1* and *BRCA2* genes in King's syndrome. 3 5 Thus, polygenetic assessment using next-generation sequencing (NGS) has been the most suitable method for investigating genetic variants associated with cancer risk, in addition to presenting cost and analysis time efficiency. 6

The criteria used to select patients at risk for hereditary cancer are often based on self-reported family history. 6 However, the absence of supporting documents (medical records, death certificate) and inaccurate information reported by patients can reduce the reliability of the information and thus restrict the use of this tool in clinical practice and in decision-making on primary surveillance recommendations and preventive measures interventions. 7 However, in Brazil, researches suggested that for middle- and low-income individuals, the use of this information, even without supporting documents, has been useful for screening patients with hereditary cancer. 8

In this study, we selected a sample of cancer patients from Monte Santo, a municipality located in the northeast region of Brazil. This city is characterized by a low level of education, a situation of extreme poverty, a low rate of immigration, a high rate of inbreeding, and consanguinity. 9 In addition, in Monte Santo, it was observed that some rare genetic diseases have a high frequency, for example, mucopolysaccharidosis type VI (1:5,000 in Monte Santo, reaching 1:1,500,000 worldwide). 10 Due to the characteristics of this population, we started a community genetics project that includes a census of diseases with probable genetic etiologies in the population, including cancer. Thus, the objective of this work was to investigate the mutational profile of patients with suspected hereditary cancer using a multigene panel through NGS.

## Materials and Methods

### Sample

During the period from August 2014 to June 2016, 116 patients diagnosed with any type of cancer in Monte Santo, Bahia (northeast Brazil) were included in the study. Of these, 30 patients with hereditary cancer characteristics were selected using the criteria: family history for the disease and age of cancer occurrence before 50 years, 11 in addition, the patients should not be related. For all participants, a questionnaire was filled out with personal and clinical data and race information by self-report according to the criteria of the Brazilian Institute of Geography and Statistics. This study was approved by the Ethics Committee of the Hospital University Prof Edgar Santos, and all subjects provided informed consent.

### Multigene Panel

The panel was composed of 17 genes, which: *BRCA1* , *BRCA2* , *APC* , *TP53* , *PTEN* , *RET* , *VHL* , *RB1* , *CDKN2* , *CDH1* , *CHECK2* , *MLH1* , *MSH2* , *MSH6* , *MUTYH* , *XPA* , and *XPC* . These genes were selected because they are associated with different cancer types and hereditary syndromes. DNA sample was obtained from 200 µL of peripheral blood using the Mini Spin Plus Extraction Kit (Biometrix, BioPur, Curitiba, Paraná, Brazil) according to the manufacturer's instructions. All DNA samples were quantified by spectrophotometry in a NanoDrop 2000c Spectrophotometer device (ThermoScientific, Wilmington, Delaware, United States) at 260/280 nm wavelengths. DNA integrity was verified in a 2.5% agarose gel and subsequently diluted to a concentration of 25 ng/µL. The panel used was TruSeq Custom Amplicon v1.5 on the MiSeq System (Illumina, San Diego, California, United States). The variants were classified according to the ClinVar database into: pathogenic, variant of uncertain significance (VUS), and of pharmacogenetic importance ( Supplementary Table S1 ).

## Results

Fifteen patients (4 women and 11 men), of the 30 who were selected, were possible to carry out the multigene panel. Most women had breast cancer (3/5) and among men, prostate cancer (2/11), and skin cancer (2/11) were the most frequent ( Table 1 ). The mean age at diagnosis was 47.8 years ± 12.87, and among those with a family history, it was 51.2 and without a family history, 41 years.

**Table 1:** General characteristics of patients with hereditary cancer profile in the Monte Santo, Bahia

Patient	Tumor site	Gender	Age at diagnosis (y)	SR	Family history a
1	Stomach	M	47	White	No
2	Skin (squamocellular)	M	41	Brown	No
3	Breast	F	39	Brown	No
4	Rectum	F	66	White	Yes
5	Prostate	M	52	Brown	Yes
6	Breast	F	44	White	Yes
7	Prostate	M	60	Brown	Yes
8	Skin (melanoma)	M	43	White	Yes
9	Breast	F	50	Brown	No
10	Breast	F	50	Brown	Yes
11	Breast	F	28	Brown	No
12	Intestine	M	69	Brown	Yes
13	Brain	M	25	Brown	Yes
14	Thyroid	F	40	Brown	Yes
15	Prostate	M	63	Black	Yes

Abbreviation: SR, self-report.

a Supplementary Table S3 .

Seven clinically important variants were found in 12 patients (58%) ( Table 2 ). Of these, 11 patients (73.3%) had some variant of pharmacogenetic importance and 1 patient (6.6%) has a pathogenic variant. All variants found were in heterozygosity. Four VUS were identified in four (26.6%) patients in the genes: *APC* , *BRCA2* , and *MUTYH* . One of the patients had two different VUS ( Supplementary Table S2 ).

**Table 2:** Variants observed in susceptibility genes evaluated by the hereditary cancer panel in patients from Monte Santo, Bahia

Gene	Variant position	Variant description	Protein alteration	Classification	Patients ( *N* )
*APC*	Chr5: 112102097	c.210G > C	p.Glu70Asp	VUS	1
*TP53*	Chr17: 7579472	c.215C > G	p.Pro72Arg	Pharmacogenetics B	5
*XPC*	Chr3: 14187449	c.2815C > A	p.Gln939Lys	Pharmacogenetics A	9
*MUTYH*	Chr1: 45797228	c.1187G > A	p.Gly396Asp	Pathogenic	1
*BRCA2*	Chr13: 32910773	c.2281T > C	p.Tyr761His	VUS	1
*MUTYH*	Chr17: 45800182	c.38C > T	p.Ala13Val	VUS	1
*APC*	Chr5: 112176905	c.5614G > A	p.Val1872Ile	VUS	1

Abbreviation: VUS, variant of uncertain significance.

Notes: Pharmacogenetics A: cisplatin toxicity. Pharmacogenetics B: cisplatin, cyclophosphamide, Fluoratil, and paclitaxel toxicity.

**Fig. 1:**
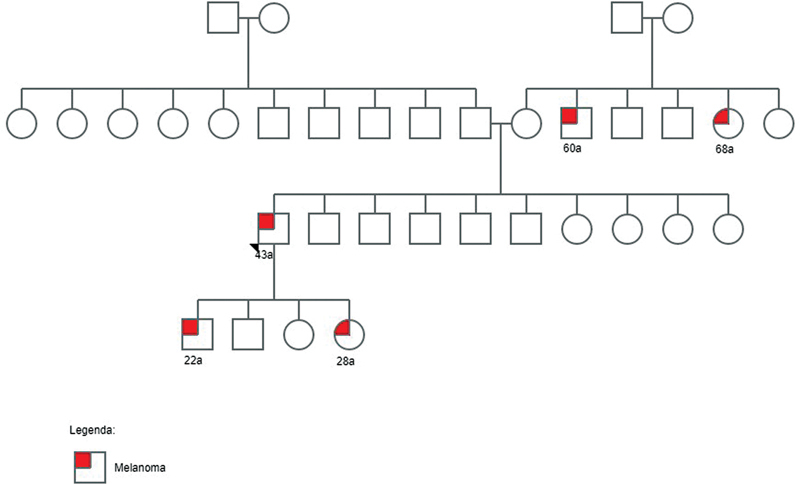
Pedigree of patient 8 with a family history of melanoma.

Each VUS was found in only one patient. For variants with pharmacogenetic importance, most patients (9/15) had the *XPC* :c.2815C > A variant (p.Gln939Lys) and the others (5/15) had the *TP53* :c.215C > G variant (p.Pro72Arg). The pathogenic variant *MUTYH* :c.1187G > A (p.Gly396Asp) was found in a male patient diagnosed with melanoma at the age of 43 years and with a family history of this tumor. The pedigree of the family history is shown in Fig. 1 .

## Discussion

In the analyzed sample, 1/16 (6.25%) patients with pathogenic mutation and 4/16 (25%) patients with VUS were identified. In a study carried out using a multigene panel with 12 genes for different cancer types and using the same criteria for cases selection in the present study (age <50 years and/or family history), 12.3% of patients had a pathogenic mutation and 19.4% patients with VUS. 12

Two pharmacologically important polymorphisms were found: p.(Gln939Lys) in the *XPC* gene (in 9/15 participants) and p.(Pro72Arg) in the *TP53* gene (5/15 participants). According to the 1000 Genomes Project, the *XPC* mutation p.(Gln939Lys) has a high frequency of the T allele in parental populations: African (0.7315) and European (0.5421) (no data in Amerindian population). The *TP53* p.(Pro72Arg) mutation, on the other hand, has a variable frequency of the G allele between African (0.6389) and European (0.2757) populations (no data in Amerindian population). The results observed are in agreement with what is expected for a mixed population as Brazil, especially the northeast Brazilian, which has a high African and European ancestral contribution. 13 14 Due to the lack of data on the treatment performed by the patients, it was not possible to use the information on the pharmacogenetically important markers found and the effect on the therapeutic approach used.

For the *MUTYH* gene, more than 300 mutations have been described according to the Leiden Open Variation Database variant database. 15 Among them, the missense mutation found in this study p.(Gly396Asp) described as pathogenic by ClinVar ( https://preview.ncbi.nlm.nih.gov/clinvar/variation/5294/ ). This amino acid is located in the C-terminal domain of the protein (exon 13) and its replacement reduces the interaction between enzyme and substrate, compromising enzymatic activity. 16 17 This is one of the main variants that predispose to MUTYH-associated polyposis (MAP), 18 a hereditary syndrome whose main clinical manifestations are the early development of multiple adenomatous polyps along the intestine and colorectal cancer (CRC). However, in addition to these, other extraintestinal manifestations can occur in patients with MAP, including skin findings (benign and malignant). 19 20 In a cohort of patients with MAP followed up, the occurrence of extraintestinal tumors and skin cancer was the second most common cancer reported, with a significant incidence (standardized incidence ratio: 2.8; 95% confidence interval: 1.5–4.8). In that study, of the 13 patients with skin cancer, 5 had the p.Gly396Asp mutation and of these, 2 were diagnosed with melanoma. 20 In contradiction, another study found no association between the risk of melanoma development or aggressiveness and this variant. 21

The *MUTYH:* p.(Gly396Asp) mutation has a higher frequency in populations of Caucasian origin (0.0089 Europeans, 0.0043 Amerindians, 0.0000 Africans) according to the 1000 Genome, and its origin has been estimated at about 6,000 to 9,000 years B.C. 22 This finding is in agreement with the present study that found the mutation in a patient with phenotypic characteristics of Europeans and with self-reported as white. In Brazil, this variant has already been described in two different studies: 3/60 patients with clinical criteria for MAP, one of the cases being homozygous 23 and 1/23 mutation-positive patients with < 100 polyps. 24 Thus, finding the mutation in 1/16 patients without criteria for MAP, but with a family history of melanoma reinforces the association of mutations in the *MUTYH* gene with other types of tumors. 25

It is important to consider the presence of *MUTYH:* p.(Gly396Asp) mutation in a patient with melanoma to justify the tumor origin, these data can be supported by the studies presented below. In principle, it is plausible to consider that epithelial tissue is exposed to the action of reactive oxygen species after ultraviolet (UV) exposure and that the presence of an efficient repair mechanism in this tissue is necessary. In fact, it has already been shown that the origin of melanoma may be related to oxidative damage specifically due to the presence of 8-oxoG molecules. 26 27 Thus, the defect in enzymes that act in the base excision repair mechanism may be important in the understanding of this tumor; however, *MUTYH* does not act in isolation. For example, skin cancer susceptibility has been demonstrated in *OGG1* -knockout mice with 8-oxoG production in the genetic material of UVB-exposed epidermal cells. 28 The *OGG1* gene encodes the 8-oxoG DNA glycosylase enzyme (OGG1) that recognizes and removes 8-oxoG, preventing future base mismatches. The involvement of the *MUTYH* gene in the development of melanoma was suggested in the study by Ogbah et al (2012) 29 after evaluating different cell lines of this type of cancer through multiplex ligation-dependent probe amplification probes that identified loss of gene heterozygosity. Furthermore, a relationship between altered *MUTYH* and *OGG1* function was observed with various tumors: neuroendocrine intestinal cells in humans, as well as risk of lung, hepatocellular and cervical cancer. 30 31 In fact, according to the Human Protein Atlas, the MUTYH protein is expressed in epithelial tissue as well as in melanoma, although in smaller amounts when compared with other tissues of the digestive system (stomach, duodenum, colon, and rectum). 32 On the contrary, OGG1 is highly expressed, both in epithelial cells and in melanoma cells, probably due to its preventive effect and prior to the action of MUTYH. Mutations in *MUTYH* may contribute to carcinogenesis, as the protein acts to prevent mutagenesis, it activates a programmed cell death pathway triggered by the 8-oxoG accumulation in nuclear and mitochondrial DNA through the activation of the Ca ^2+^ -dependent protease, calpain. 33 It was later shown that this pathway could still be PARP/MLH1 dependent mediating the activity of p53, tumor suppressor protein. 34 It is already known that downregulation of calpain-3 and MLH1 inactivation are events that contribute to the progression of melanoma, reinforcing the importance of *MUTYH* functionality. 35 36 37 So, in the absence of MUTYH, such premutagenic or precancerous cells could survive and have a higher mutation rate in proto-oncogenes or tumor suppressor genes due to increased levels of 8-oxoG.

It is interesting to consider that the pathogenic mutations in MUTYH associated with hereditary cancer have been mostly reported in homozygosity, with the complete inactivation of its product. However, it has been suggested that the risk of developing CRC is higher in both heterozygous and homozygous *MUTYH* mutant individuals than in individuals without pathogenic alleles, even those with a family history of CRC. 38 In a study with neuroendocrine tumors of the small intestine, in which the presence of the p.Gly396Asp mutation was detected in heterozygosity in 6/24 patients with and without a family history, it was suggested that the biallelic inactivation of *MUTYH* may not be the only mechanism that drove tumor development and that additional mutations in *OGG1* would be important for the pathogenesis of the disease. 31 In view of the presented data and the available literature, it is possible to suggest that the presence of a mutation in the *MUTYH* gene in heterozygosis may be responsible for a less aggressive phenotype, with late onset and slow tumor progression. In the literature, an association has already been observed between high risk for breast cancer and the variant found in the present study in heterozygosis. 39

It is important to emphasize that the patient with a pathogenic mutation in the present study has a family history of melanoma and that no alterations were found in *CDKN2* , the gene responsible for susceptibility in approximately 22% of familial cases with a single mutation and in more than half of the individuals diagnosed with multiple primary melanomas. 40 With this, it is still possible to suggest that other genes of lesser relevance are involved in the disease process, such as the *OGG1* gene.

## Conclusion

The results of this study suggest that through the selection of patients with a hereditary cancer profile, it was possible to identify mutations of clinical importance, such as those of pharmacogenetic importance, of uncertain significance (VUS) and pathogenic mutations, even in a small sample of individuals. The use of a multigenic panel made it possible to identify the pathogenic mutation *MUTYH* p.(Gly396Asp) in a patient with melanoma and a family history. Mutations in this gene have been poorly studied in patients with this neoplasm, but the biological plausibility indicates that there is evidence for this association and thus justifies the expansion of the study. This is also an advantage of multiple gene analysis, where new associations can be identified.
