# Supplementary material for: Screening for Mutations in Hereditary Cancer Susceptibility Genes in a Region with High Endogamy in Brazil
Source: Glob Med Genet. 2023 Dec 8;10(4):376–81. doi: 10.1055/s-0043-1777449 (PMC10709072; doi:10.1055/s-0043-1777449)
Supplement: Supplementary file 1 — Supplementary Material [file 10-1055-s-0043-1777449-s2300074.pdf]

Supplementary Table S1 Major hereditary cancer syndromes and their associated genes

Gene	Location	Function	Syndromes and neoplasms
BRCA1	17q	Tumor suppressor	HBOC/King's syndrome
BRCA2	13q	Tumor suppressor	HBOC/King's syndrome
hMSH2	2p16	DNA repair	Lynch's syndrome
hMLH1	3p21	DNA repair	Lynch's syndrome
*APC*a	5q22	Tumor suppressor	Familial adenomatous polyposis
TP53	17p13	Tumor suppressor	Li–Fraumeni's syndrome
PTEN	10q22-23	Tumor suppressor	Cowden's syndrome and Bannayan–Riley–Ruvalcaba
RET	10q	Oncogene	Multiple endocrine neoplasia type 2A or 2B
VHL	3p25-26	Tumor suppressor	Von Hippel–Lindau's disease
RB1	13q14	Tumor suppressor	Hereditary retinoblastoma
CDKN2	9p21	Tumor suppressor	Familial melanoma
CDH1	16q22.1	Tumor suppressor	Hereditary diffuse gastric cancer
CHEK2	22q12.1	Tumor suppressor	HBOC/King syndrome
MSH6	2p16	Tumor suppressor	Lynch's syndrome
MUTYH	1p34.1	Tumor suppressor	Polyposis, colorectal cancer
XPA	9q34.1	Tumor suppressor	Xeroderma pigmentosum (skin cancer)
XPC	3p25.1	Tumor suppressor	Xeroderma pigmentosum (skin cancer)

Abbreviation: HBOC, hereditary breast and ovarian cancer syndrome.
Source: National Family Cancer Network: Operational Manual/Instituto Nacional de Câncer—Rio de Janeiro: INCA, Brazilian National Cancer Institute, Ministry of Health, 2009.

 © 2023. The Author(s).

Supplementary Table S2 Pathogenic variants, of pharmacogenetic importance and VUS, identified in patients with a hereditary cancer profile in the population of Monte Santo, Bahia

Patient	Gene	Variant position	Variant	Protein alteration	Classification
1	Unidentified variants				
2	*APC*	Chr5: 112102097	c.210G > C	p.Glu70Asp	VUS
	TP53	Chr17: 7579472	c.215C > G	p.Pro72Arg	Pharmacogenetics B
3	Unidentified variants				
4	*XPC*	Chr3: 14187449	c.2815C > A	p.Gln939Lys	Pharmacogenetics A
5	Unidentified variants				
6	*XPC*	Chr3: 14187449	c.2815C > A	p.Gln939Lys	Pharmacogenetics A
	TP53	Chr17: 7579472	c.215C > G	p.Pro72Arg	Pharmacogenetics B
7	*XPC*	Chr3: 14187449	c.2815C > A	p.Gln939Lys	Pharmacogenetics A
8	*MUTYH*	Chr1: 45797228	c.1187G > A	p.Gly396Asp	Pathogenic
9	*XPC*	Chr3: 14187449	c.2815C > A	p.Gln939Lys	Pharmacogenetics A
10	*BRCA2*	Chr13: 32910773	c.2281T > C	p.Tyr761His	VUS
	XPC	Chr3: 14187449	c.2815C > A	p.Gln939Lys	Pharmacogenetics A
	TP53	Chr17: 7579472	c.215C > G	p.Pro72Arg	Pharmacogenetics B
11	*TP53*	Chr17: 7579472	c.215C > G	p.Pro72Arg	Pharmacogenetics B
12	*XPC*	Chr3: 14187449	c.2815C > A	p.Gln939Lys	Pharmacogenetics A
13	*TP53*	Chr17: 7579472	c.215C > G	p.Pro72Arg	Pharmacogenetics B
	XPC	Chr3: 14187449	c.2815C > A	p.Gln939Lys	Pharmacogenetics A
14	*XPC*	Chr3: 14187449	c.2815C > A	p.Gln939Lys	Pharmacogenetics A
15	*MUTYH*	Chr17: 45800182	c.38C > T	p.Ala13Val	VUS
	APC	Chr5: 112176905	c.5614G > A	p.Val1872Ile	VUS
	XPC	Chr3: 14187449	c.2815C > A	p.Gln939Lys	Pharmacogenetics A

Abbreviation: VUS, variant of uncertain significance.
Notes: Pharmacogenetics A: cisplatin toxicity. Pharmacogenetics B: cisplatin, cyclophosphamide, Fluoratil and paclitaxel toxicity.

 © 2023. The Author(s).

Supplementary Table S3 Family history of patients with hereditary cancer profile in the population of Monte Santo, Bahia

Pct	Cancer	Gender	DA	SR	Familial history
1	Stomach	M	47	White	No
2	Skin (Sq)	M	41	Brown	No
3	Breast	F	39	Brown	No
4	Rectal	F	66	White	Niece (35 y)—stomach
5	Prostate	M	52	Brown	Father (78 y)—pancreas<br>Maternal aunt (?)—breast<br>Two maternal cousins (35 y;17 y)—breast<br>Paternal aunt (60 y)—leukemia<br>Paternal aunt (50 y)—uterus<br>Paternal cousin (35 y)—throat
6	Breast	F	44	White	Four second degree cousins (26 y; 29 y; 30 y; 39 y)—breast<br>Father (78 y)—prostate
7	Prostate	M	60	Brown	Paternal uncle (60 y)—prostate<br>Paternal Aunt (70 y)—breast<br>Two siblings (?)—prostate
8	Skin (Ml)	M	43	White	Maternal uncle (60 y)—skin<br>Maternal uncle (68 y)—skin<br>Mother (69 y)—skin<br>Son (22 y)—skin<br>Son (28 y)—skin
9	Breast	F	50	Brown	No
10	Breast	F	50	Brown	Father (80 y)—prostate<br>Maternal uncle (70 y)—prostate<br>Maternal cousin (?)—prostate
11	Breast	F	28	Brown	No
12	Colon	M	69	Brown	Mother (?)—head
13	Brain	M	25	Brown	Maternal aunt (60 y)—colon<br>Maternal cousin (?)—?<br>Maternal aunt (?)—liver
14	Thyroid	F	40	Brown	Father (?)—prostate<br>Mother (?)—skin<br>Paternal aunt (?)—lung<br>Paternal grandmother (?)—skin<br>Paternal cousin (?)—testicle<br>Paternal cousin (?)—breast<br>Maternal cousin (?)—uterus
15	Prostate	M	63	Black	Maternal grandfather, uncle, and cousin (?)—prostate

Abbreviations: DA, diagnosis age; Pct, patient; Skin (Ml), skin cancer (melanoma); Skin (Sq), skin cancer squamocellular; SR, self-reported race/color (IBGE).

 © 2023. The Author(s).
